# Research on a bifurcation location algorithm of a drainage tube based on 3D medical images

**DOI:** 10.1186/s42492-019-0039-0

**Published:** 2020-01-14

**Authors:** Qiuling Pan, Wei Zhu, Xiaolin Zhang, Jincai Chang, Jianzhong Cui

**Affiliations:** 1 College of Sciences, North China University of Science and Technology, Tangshan 063210, Hebei China; 2 Department of Neurosurgery, Tangshan Gongren Hospital, Tangshan 063000, Hebei China

**Keywords:** Multitube drainage tube, Bifurcation localization algorithm, 3D medical image, Path planning, Intracranial hematoma

## Abstract

Based on patient computerized tomography data, we segmented a region containing an intracranial hematoma using the threshold method and reconstructed the 3D hematoma model. To improve the efficiency and accuracy of identifying puncture points, a point-cloud search arithmetic method for modified adaptive weighted particle swarm optimization is proposed and used for optimal external axis extraction. According to the characteristics of the multitube drainage tube and the clinical needs of puncture for intracranial hematoma removal, the proposed algorithm can provide an optimal route for a drainage tube for the hematoma, the precise position of the puncture point, and preoperative planning information, which have considerable instructional significance for clinicians.

## Introduction

Trauma can cause blood vessels to burst in the brain or between the skull and brain tissue. Subsequently, an intracranial hematoma may form from blood pooling up in the brain or between the skull and brain, which compresses the brain tissue. An intracranial hematoma is a common but serious secondary damage mode of craniocerebral injury. The incidence of closed craniocerebral injury is approximately 10%, while the incidence of severe craniocerebral injury is approximately 40%–50%. The complications of intracranial hematomas include their effect on cerebral blood flow, cerebral hernia, cerebral edema, and Cushing’s reaction, which can seriously damage brain tissue, and some of these damage modes are irreversible and life-threatening [1–4]. Puncture and ablation of the intracranial hematoma is a widely used medical method. With the increasingly busy modern life, heavy work stress, mental pressure, and lack of physical labor, the incidence rates show that the age of onset of this condition is decreasing. Once it occurs, the disease places a burden on individuals and families, so all countries and the international community have attached considerable importance to it [2]. The advantages of puncture operation include effective application, high security, quick recovery, short operating time, and relatively few postoperative complications [5–7]. At present, the methods of positioning such as the stereotactic technique [8] and neuronavigation technique [9] mainly involve a freehand technique for insertion of a drainage tube, which is based on fixed anatomical landmarks, does not consider individual variations, and often exhibits insufficient precision. Hence, a time-efficient and low-cost technique to localize the hematoma puncture point and to provide path planning will be beneficial, especially when highly sophisticated and expensive navigation systems cannot be made available in developing regions. In the present study, according to a dataset of brain computerized tomography (CT) images, we reconstructed the 3D model of a patient’s brain by using a 3D Slicer software [10–14] and extracted the area of intracranial hematoma as well. To quickly find the best puncture point in theory, an algorithm based on k-means clustering [15] was proposed to optimize the search space, eliminate redundant computation, effectively sort out the search space, and reduce the possibility of particles falling into a local extremum [16]. Then, the algorithm is improved from the viewpoint of the search mode, and the point cloud search algorithm of adaptive weighted particle swarm optimization [17–19] is proposed, which greatly reduces the calculation time and the number of iterations of the algorithm. In the experimental environment, the algorithm proposed in this study can greatly improve the search efficiency of this method in obtaining the global optimal solution. Compared with the direct optimization algorithm [20], the average maximum acceleration ratio is 1288.67%. According to the characteristics of the multitube drainage tube [21] and the clinical needs of the puncture process for intracranial hematoma removal, the bifurcation location algorithm provides the precise position of the puncture point, the optimum location of probes, and the best route under ideal conditions. In this study, the 3D model is effectively combined with the multitube drainage tube; furthermore, a preoperative simulation is proposed, which can provide significant guidance and value in formulating the puncture operation plan and decreasing the risk of blindness.

## Segmentation of the intracranial hematoma

In clinical medicine, for patients with an intracranial hematoma, most hospitals conduct CT, hematoma location, puncture marking, followed by other processes [5, 6]. We used the 3D Slicer software to implement three-dimensional reconstruction based on brain CT data. This approach can greatly reduce the treatment time and improve efficiency. 3D Slicer software can reconstruct anatomical parts such as the hematoma, blood vessels, skull, and nerve bundles. In this way, doctors can avoid important parts such as blood vessels and nerve bundles when they mark the puncture points and accurately and efficiently puncture the hematoma, thus reducing treatment time [12–14]. The 3D Slicer program is a software platform for the analysis (including registration and interactive segmentation) and visualization (including volume rendering) of medical images and for research in image guided therapy. The platform originated in an MSc thesis program in 1998 and is jointly sponsored by the Surgery Plan Laboratory at Brigham and Women’s Hospital and the Artificial Intelligence Laboratory at the Massachusetts Institute of Technology [10–12].

### Hematoma modeling

After the CT data have been preprocessed, three-dimensional reconstruction of the brain can be realized to obtain a three-dimensional image. In 3D Slicer, the region of interest (ROI) function is used to extract the hematoma area in the image, and the Segmentations module is selected to model the hematoma, providing models in the STL and OBJ formats to extract the “optimal external central axis” needed in this study.

### Hematoma location locking

**Fig. 1:**
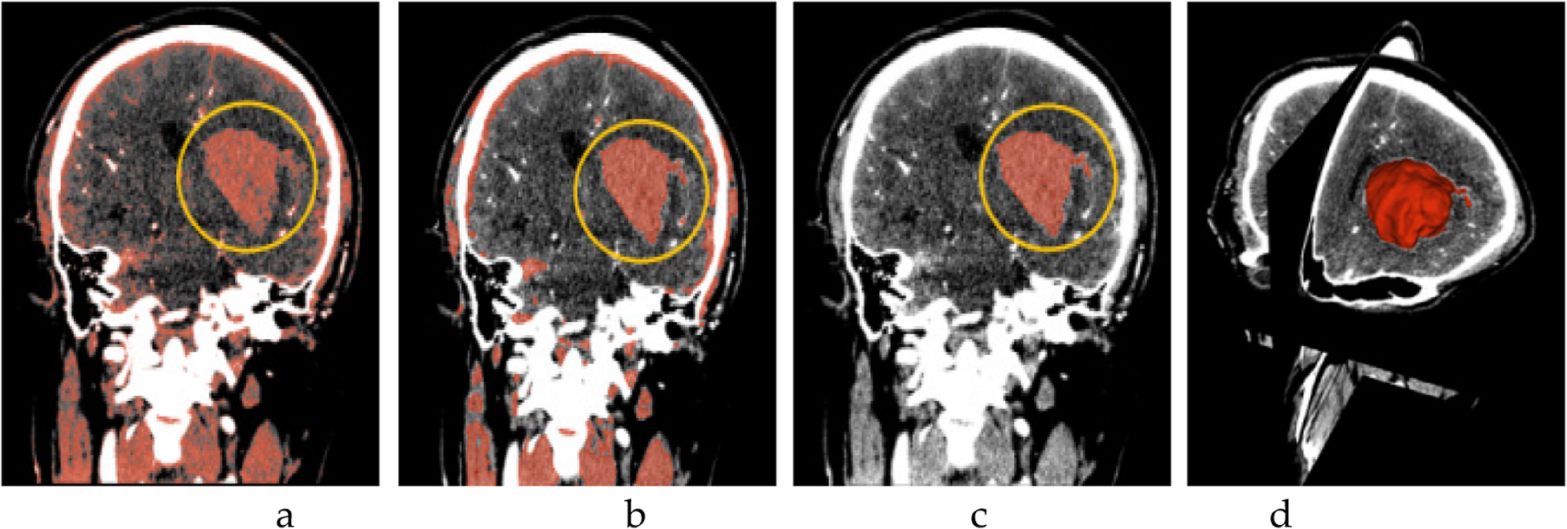
Process of distinguishing the hematoma in the yellow circle. **a** Thresholding; **b** Using smoothing to optimize staining; **c** Using Islands to separate the hematoma; and **d** The fusion of the hematoma and the three-dimensional map

First, the Paint function in Slicer is applied to draw the hematoma, and then, the Fast Marching Method is used to draw the models of the intracerebral hematoma within a three-dimensional sphere, which is of great help in the follow-up work. Finally, the three-dimensional sphere is initialized by the Fast Marching function. Because the mask area around the hematoma is very large after initialization, the mask is narrowed by adjusting the slider for Segment volume, and the position of the hematoma is locked (Fig. 1).

### Applying the threshold method to segment the hematoma

**Fig. 2:**
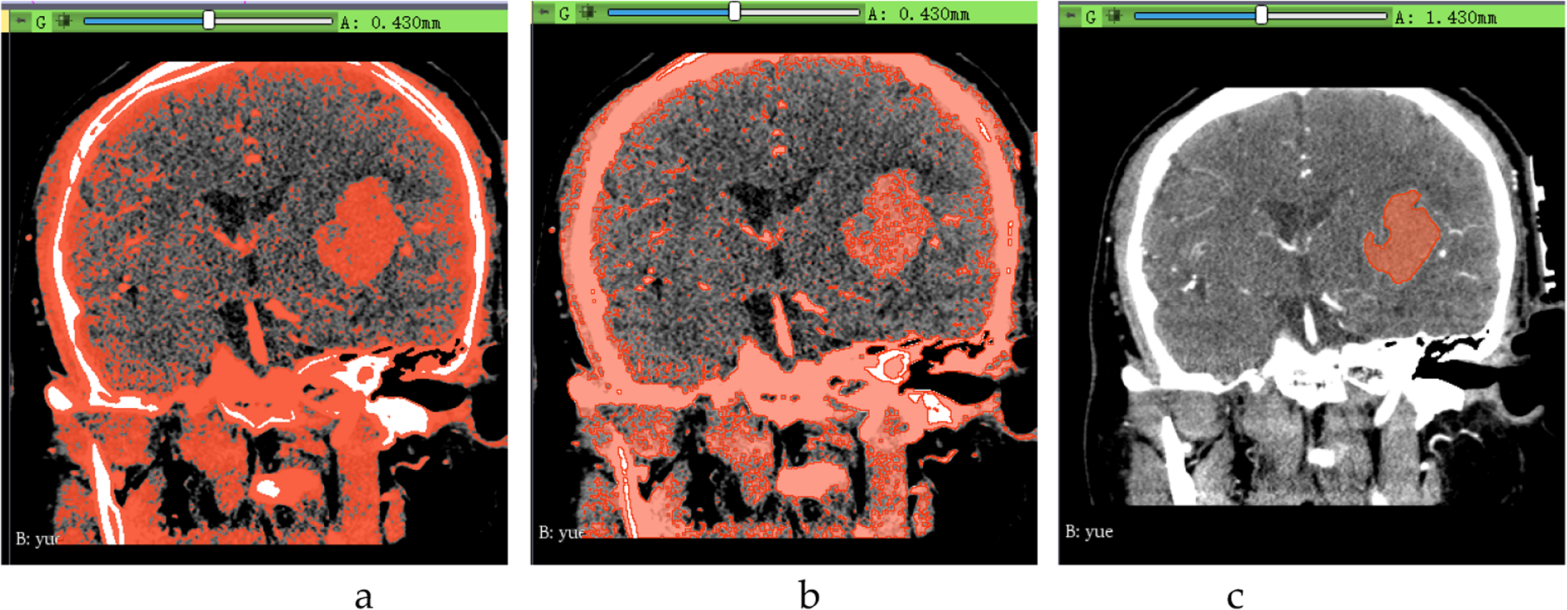
Schematic diagram of hematoma extraction. **a** Mask effect; **b** Smoothing; and **c** hematoma segmentation

We select the Threshold function, in which the threshold is set to 50–100, set the selected intensity range to an editable intensity for the mask, and then, convert the masked area into a 3D model. For multiple small masks that appear in CT images, we use the Smoothing function to make the hematoma easier to segment by removing protrusions and filling holes to make the segmentation boundaries smoother. After smoothing, we use the Islands function to segment the hematoma from the numerous masks. We select the Keep selected island and click on the hematoma to segment it (Fig. 2). For specific modeling steps and methods, please refer to ref. [12].

## Methods

### Basic concept and definition

As an effective tool to describe the morphological characteristics of the geometry, the center axis has many advantages; for example, it can describe the geometry information inside the model and at any position of the boundary [22–24]. Furthermore, it provides a method to intuitively describe the geometric characteristics of a complex model and represent the topological relation of the model. The definition of the external central axis is as follows: Let *Ω* be a model in *n*-dimensional Euclidean space *R*^*n*^, and assume it is homeomorphic to a 3D closed sphere; we define *Ω* as an *n* − *D* model. The external center axis of *Ω* is the set of the centers of the maximum circumscribed spheres [25]. Inspired by the concept of the external center axis, we found the optimal external center axis suitable for this study. The maximum Euclidean distance in the hematoma point cloud is determined, and the line segment joining the two points is defined as the optimal external center axis.

### Algorithm of optimal external central axis extraction

The optimal external central axis can be obtained using a direct optimization algorithm [20]. However, a large amount of computation is required because the time complexity increases at the N^2^ level with an increasing quantity of point clouds. In the case of a large-scale geometric model, the approach to obtain the central axis is excessively time consuming. Therefore, to reduce the computation redundancy, a point cloud clustering algorithm based on k-means is proposed to optimize the search space [15, 16]. The algorithm presents strong adaptive ability, robustness, and low computation requirements. However, it does not consider the search method, and thus, it is not a suitable method for extracting the axis. Based on the above analysis, we propose the point cloud search arithmetic method for modified adaptive weighted particle swarm optimization [17–19, 26–30]. The algorithm reduces computational redundancy and greatly enhances the efficiency of searching axes in complex geometries. Our proposed algorithm is written in MATLAB (The MathWorks, Inc., MA).

### Algorithm flow

The flow of the point cloud search arithmetic method for modified adaptive weighted particle swarm optimization based on k-means clustering is as follows:

Step 1. First, define the data set *D* in 3D Euclidean space and randomly select *n* items (*n* is the number of selected clusters) as centroids *C*_*i*_(*i* = 1, 2, ⋯, *n*), where each centroid is the clustering center of a category. Then, calculate the Euclidean distance between *C*_*i*_ and *D*_*j*_(1, 2, ⋯, *k*) in *D.* For example, if *D*_*j*_ is the nearest to *C*_*i*_, it is classified as cluster *i*.

Step 2. Through the first step, the data set *D* is initially divided into *k* classes. Calculate the mean value of each dimension of all data items in each cluster. A new centroid is formed and updated to the new centroid.

Step 3. Repeat steps 1 and 2 to obtain the new centroid until the centroid of each class no longer changes.

Step 4. Randomly initialize the particles in the solution space.

Step 5. Each particle updates its velocity and position by tracking its own and the group’s optimal solution. The velocity updating formula of particle *i* in the *d*th dimension is as follows:

1 $$ {v}_{id}^k=\omega {v}_{id}^{k-1}+{c}_1{r}_1\left( pbes{t}_{id}-{x}_{id}^{k-1}\right)+{c}_2{r}_2\left( pbes{t}_{id}-{x}_{id}^{k-1}\right) $$

The position updating formula is

2 $$ {x}_{id}^k={x}_{id}^{k-1}+{v}_{id}^{k-1} $$

Repeat step 5 and incrementally iterate until the optimal position of the given threshold is found.

Note: ${v}_{id}^k$ is the *d* − *th* dimensional component of the *k* − *th* iterative particle *i* flight velocity vector; ${x}_{id}^k$ is the *d* − *th* dimensional component of the position vector; *c*_1_*c*_2_ is the maximum learning step parameter; *r*_1_*r*_2_ is the random function in the range of [0 − 1]; and *ω* is the inertia weight of the search, which has a considerable impact on the search scope and method.

Step 6. Compute the mean distance between *d*_1_, *d*_2_, ⋯, *d*_*n*_: ${D}_{EXP}=\frac{2\ast \sum {D}_{ij}}{k\ast \left(k-1\right)}$, where ${D}_{ij}=\sqrt{{\left({x}_i-{x}_j\right)}^2+{\left({y}_i-{y}_j\right)}^2+{\left({z}_i-{z}_j\right)}^2}$ denotes the distance between two clustering centers.

Step 7. Calculate and obtain the difference between *D*_*ij*_ and *D*_*EXP*_: *S*_*ij*_ = *D*_*ij*_ − *D*_*EXP*_, where *ω*_*ij*_ denotes the ratio of *S*_*ij*_ to ∑*S*_*ij*_ as the generating weight of the small block *ω*_*ij*_: ${\omega}_{ij}=\frac{S_{ij}}{\sum {S}_{ij}}$.

**Fig. 3:**
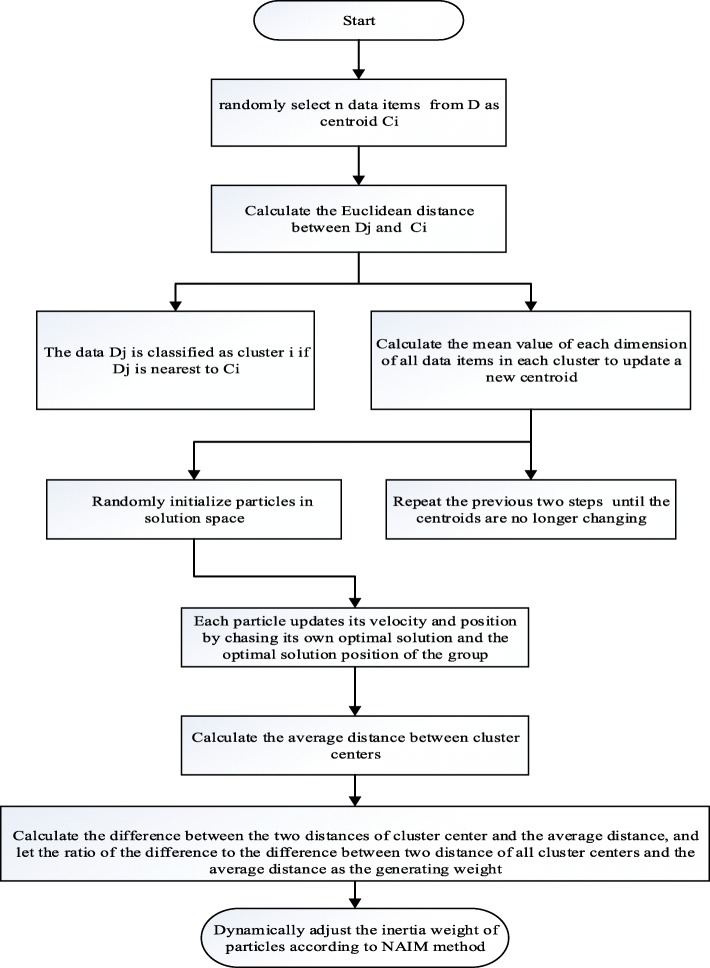
Flow diagram of the algorithm to extract the optimal external middle axis

Step 8. Dynamically adjust the inertial weight of particles according to the NAIW method proposed by Li et al. [30].

3 $$ {\omega}_i^j=k\times \frac{\omega_{\mathrm{max}}-{\omega}_{\mathrm{min}}}{\max \left\{\varDelta {x}_i^t\right\}}+{\omega}_{\mathrm{min}} $$

4 $$ \varDelta {x}_i=\sqrt{\sum_{d=1}^D{\left({x}_{id}-{G}_d\right)}^2} $$

5 $$ k=\frac{iterNum-t}{iterNum} $$

where *ω*_max_ and *ω*_min_ are 0.9 and 0.4, respectively [20], *Δx*_*i*_ is the distance between the *ith* particles and the extreme position of the group, and *k* is the iteration coefficient. The NAIW method balances the weight of the particles under different circumstances, and it has advantages in convergence and robustness. Figure 3 is the flow diagram of the algorithm.

### Optimal path planning

According to clinical demand and geometry symmetry, the straight line, where the optimal external axis is, is the path of the drainage tube into the brain. The multitube drainage tube can be divided into two subtubes and three subtube drainage tubes. The purpose of this study is to provide the corresponding optimal path planning for a specific multitube drainage tube [21]. The Table 1 is the explanation of symbols.

**Table 1:** Notation

Symbol	Description
*M*_1_(*x*_1_, *y*_1_, *z*_1_)**,** *M*_2_(*x*_2_, *y*_2_, *z*_2_)	The coordinate of the joint point of the optimal external axis and the boundary of the hematoma
*d*_12_ = ∣ *M*_1_*M*_2_∣	The distance from *M*_1_ to *M*_2_
*d*_34_ = ∣ *M*_3_*M*_4_∣	The maximum diameter of the vertical surface through *M*_1/2_ in the hematoma
*M*_3_(*x*_3_, *y*_3_, *z*_3_)**,** *M*_4_(*x*_4_, *y*_4_, *z*_4_)	The coordinates of the endpoints of *d*_34_ in the hematoma
*D***,** *d*	*D*denotes the diameter of the duct in the main tube; *d* denotes the diameter of the hole in the subtube
*d*_*O*_	The distance between the two central points of the holes
*d*_*B*_	The distance between the two bifurcations
${d}_{B_i}$	The distance between the main probe tip and the *i* − *th* bifurcation-tube probe tip (we define the first subtube to be nearest to the main probe tip)

Note: Only some common symbols are listed here; the rest are explained later

### The known conditions can be determined by *M*_1_ and *M*_2_

According to the intersection coordinates *M*_1_(*x*_1_, *y*_1_, *z*_1_) and *M*_2_(*x*_2_, *y*_2_, *z*_2_), we obtain the parameter equation of the center axis as follows:

$$ \Big\{{\displaystyle \begin{array}{c}x={x}_1+\left({x}_2-{x}_1\right)\ t\\ {}y={y}_1+\left({y}_2-{y}_1\right)\ t\\ {}z={z}_1+\left({z}_2-{z}_1\right)\ t\end{array}},\mathrm{where}\ 0\le t\le 1 $$ (6)

The midpoint coordinates of *M*_1_*M*_2_ are ${M}_{1/2}=\left(\frac{x_1+{x}_2}{2},\frac{y_1+{y}_2}{2},\frac{z_1+{z}_2}{2}\right)$_._

The distance from *M*_1_ to *M*_2_ is ${d}_{12}=\sqrt{{\left({x}_2-{x}_1\right)}^2+{\left({y}_2-{y}_1\right)}^2+{\left({z}_2-{z}_1\right)}^2}$_._

The plane equation of *M*_3_*M*_4_ is

7 $$ \left({x}_2-{x}_1\right)\left(x-\frac{x_1+{x}_2}{2}\right)+\left({y}_2-{y}_1\right)\left(y-\frac{y_1+{y}_2}{2}\right)+\left({z}_2-{z}_1\right)\left(z-\frac{z_1+{z}_2}{2}\right)=0 $$

The distance from *M*_3_ to *M*_4_ is ${d}_{34}=\sqrt{{\left({x}_4-{x}_3\right)}^2+{\left({y}_4-{y}_3\right)}^2+{\left({z}_4-{z}_3\right)}^2}$_._

A comparison of the distance between *d*_12_ and ${d}_{B_i}$ can offer directional evidence for puncture surgery.

8 $$ \Big\{{\displaystyle \begin{array}{cc}\frac{d_{B_3}-{d}_{B_2}}{2}+{d}_{B_2}<{d}_{12},& \mathrm{select}\ \mathrm{three}-\mathrm{subtubes}\ \\ {}\frac{d_{B_3}-{d}_{B_2}}{2}+{d}_{B_2}\ge {d}_{12},& \mathrm{select}\ \mathrm{two}-\mathrm{subtubes}\end{array}} $$

**Fig. 4:**
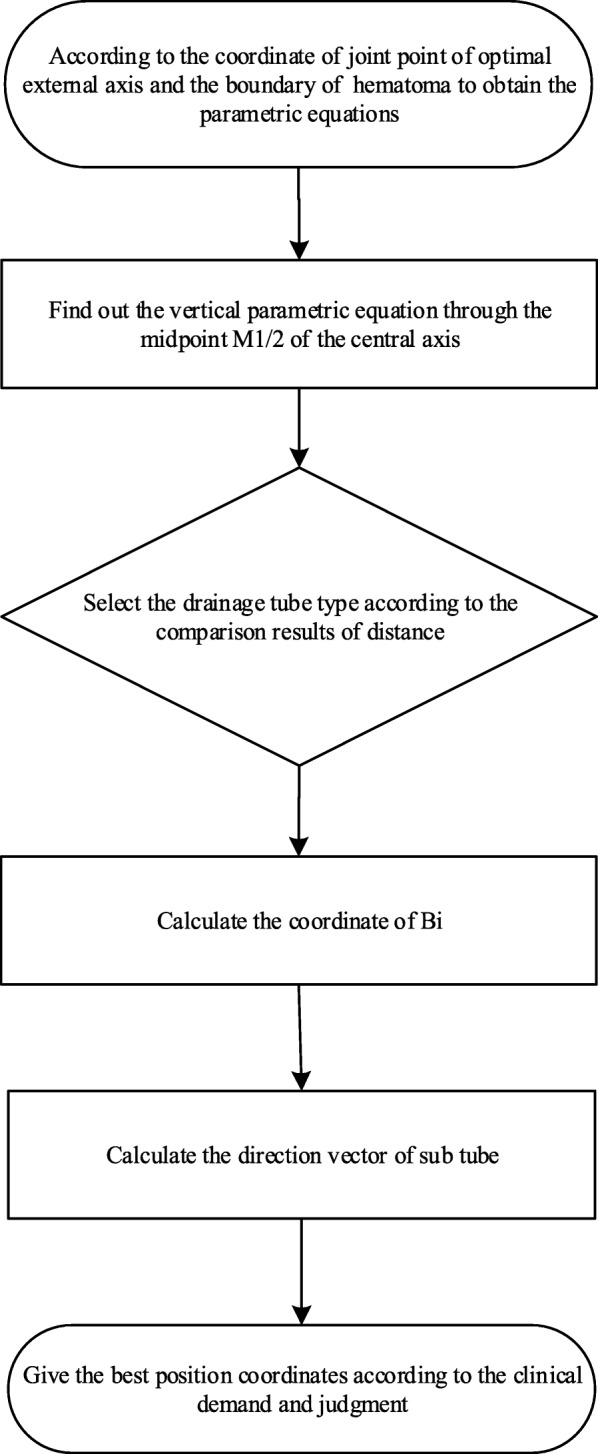
Flow diagram of the bifurcation location algorithm of the drainage tube

The flow diagram of the bifurcation location algorithm of the drainage tube is as shown in Fig. 4.

### Optimal path planning of two subtube drainage tubes

According to the clinical demand, the best position of the drainage tube in the hematoma is the midpoint between the two holes, coinciding with the midpoint of the central axis, that is, ${M}_{1/2}=\left(\frac{x_1+{x}_2}{2},\frac{y_1+{y}_2}{2},\frac{z_1+{z}_2}{2}\right)$, and the path of the main drainage tube coincides with the optimal external center axis.

#### Calculating the coordinates of *B*_1_ and *B*_2_

9 $$ {t}_1=\frac{\mid {M}_{1/2}{B}_1\mid +\mid {M}_{1/2}{M}_1\mid }{\mid {M}_1{M}_2\mid }=\frac{d_{12}+{d}_O-d-D}{2{d}_{12}} $$

10 $$ {t}_2=\frac{\mid {M}_1{B}_1\mid +\mid {B}_1{B}_2\mid }{\mid {M}_1{M}_2\mid }=\frac{d_{12}+{d}_O+2{d}_B-d-D}{2{d}_{12}} $$

Substituting *t*_1_, *t*_2_ into Eq. (6), we obtain the coordinates of ${B}_1\left({x}_{B_1},{y}_{B_1},{z}_{B_1}\right)$ and ${B}_2\left({x}_{B_2},{y}_{B_2},{z}_{B_2}\right)$_._

#### Calculating the direction vector of the subtube

To find two line segments through *M*_3_ with a fixed angle between this axis and the center axis, let the intersection points between the line segment and the axis be *M*_31_, *M*_32_; these points should meet the following limits:

11 $$ \Big\{{\displaystyle \begin{array}{c}\frac{\overrightarrow{M_{31}{M}_3}\cdot \overrightarrow{M_1{M}_2}}{\mid \overrightarrow{M_{31}{M}_3}\mid \ast \mid \overrightarrow{M_1{M}_2}\mid }=-\frac{\sqrt{2}}{2}\\ {}\frac{\overrightarrow{M_{32}{M}_3}\cdot \overrightarrow{M_1{M}_2}}{\mid \overrightarrow{M_{32}{M}_3}\mid \ast \mid \overrightarrow{M_1{M}_2}\mid }=\frac{\sqrt{2}}{2}\\ {}\overrightarrow{M_{31}{M}_3}\cdot \overrightarrow{M_{32}{M}_3}=0\\ {}{t}_{31}=\frac{d_{12}+2\mid {M}_{1/2}{M}_3\mid }{2{d}_{12}}\\ {}{t}_{32}=\frac{d_{12}-2\mid {M}_{1/2}{M}_3\mid }{2{d}_{12}}\end{array}} $$

Substituting *t*_31_, *t*_32_ into Eq. (6), we obtain the coordinates of *M*_31_, *M*_32_; the direction vector of subtubes are $\overrightarrow{M_{31}{M}_3}=\left({m}_{31},{n}_{31},{k}_{31}\right)$ and $\overrightarrow{M_3{M}_{32}}=\left({m}_{32},{n}_{32},{k}_{32}\right)$, and the parameter equation of the two subtubes is as follows:

Scenario one:

$$ \left\{{\displaystyle \begin{array}{c}x={x}_{B_1}+{m}_{31}l\\ {}y={y}_{B_1}+{m}_{31}l\\ {}z={z}_{B_1}+{m}_{31}l\end{array}},\right\{{\displaystyle \begin{array}{c}x={x}_{B_2}+{m}_{32}l\\ {}y={y}_{B_2}+{m}_{32}l\\ {}z={z}_{B_2}+{m}_{32}l\end{array}} $$ (12)

Scenario two:

$$ \left\{{\displaystyle \begin{array}{c}x={x}_{B_1}+{m}_{32}l\\ {}y={y}_{B_1}+{m}_{32}l\\ {}z={z}_{B_1}+{m}_{32}l\end{array}},\right\{{\displaystyle \begin{array}{c}x={x}_{B_2}+{m}_{31}l\\ {}y={y}_{B_2}+{m}_{31}l\\ {}z={z}_{B_2}+{m}_{31}l\end{array}},\left(\mathrm{where}\ l\ge 0\right) $$ (13)

The intersection points between the four rays and the boundary of the hematoma are *B*_11_, *B*_21_, *B*_12_, and *B*_22_.

14 $$ \Big\{{\displaystyle \begin{array}{ccc}\mid {B}_1{B}_{11}\mid +\mid {B}_2{B}_{21}\mid \ge \mid {B}_{12}{B}_1\mid +\mid {B}_{22}{B}_2\mid & & \mathrm{scenario}\kern0.5em \mathrm{one}\\ {}\mid {B}_1{B}_{11}\mid +\mid {B}_2{B}_{21}\mid <\mid {B}_{12}{B}_1\mid +\mid {B}_{22}{B}_2\mid & & \mathrm{scenario}\kern0.5em \mathrm{two}\end{array}} $$

In the first scenario, the coordinates of the probe tip of the subtube are as follows:

$$ {B}_1^F=\left(\frac{x_{B_1}+{x}_{B_{11}}}{2},\frac{y_{B_1}+{y}_{B_{11}}}{2},\frac{z_{B_1}+{z}_{B_{11}}}{2}\right),{B}_2^F=\left(\frac{x_{B_2}+{x}_{B_{21}}}{2},\frac{y_{B_2}+{y}_{B_{21}}}{2},\frac{z_{B_2}+{z}_{B_{21}}}{2}\right) $$ (15)

In the second scenario, the coordinates of the probe tip of the subtube are.

$$ {B}_1^F=\left(\frac{x_{B_1}+{x}_{B_{12}}}{2},\frac{y_{B_1}+{y}_{B_{12}}}{2},\frac{z_{B_1}+{z}_{B_{12}}}{2}\right),{B}_2^F=\left(\frac{x_{B_2}+{x}_{B_{22}}}{2},\frac{y_{B_2}+{y}_{B_{22}}}{2},\frac{z_{B_2}+{z}_{B_{22}}}{2}\right) $$ (16)

### The optimal path planning of three subtube drainage tubes

According to the clinical demand, the best position of the drainage tube in the hematoma is the midpoint of the second hole, coinciding with the midpoint of the central axis, i.e., ${M}_{1/2}=\left(\frac{x_1+{x}_2}{2},\frac{y_1+{y}_2}{2},\frac{z_1+{z}_2}{2}\right)$, with the path of the main drainage tube coinciding with the optimal external center axis.

#### Calculating the coordinate of *B*_1_, *B*_2_, and *B*_3_

17 $$ {t}_{11}=\frac{\mid {M}_1{B}_1\mid }{\mid {M}_1{M}_2\mid }=\frac{d_{12}-d-D}{2{d}_{12}} $$

18 $$ {t}_{12}=\frac{\mid {M}_1{B}_2\mid }{\mid {M}_1{M}_2\mid }=\frac{d_{12}+2{d}_O-d-D}{2{d}_{12}} $$

19 $$ {t}_{13}=\frac{\mid {M}_1{B}_3\mid }{\mid {M}_1{M}_2\mid }=\frac{d_{12}+4{d}_O-d-D}{2{d}_{12}} $$

Substituting *t*_11_, *t*_12_ and *t*_13_ into Eq. (6), we obtain the coordinates ${B}_1\left({x}_{B_1},{y}_{B_1},{z}_{B_1}\right)$, ${B}_2\left({x}_{B_2},{y}_{B_2},{z}_{B_2}\right)$, and ${B}_3\left({x}_{B_3},{y}_{B_3},{z}_{B_3}\right)$.

#### Calculating the direction vector of the subtube

To find a line segment through *M*_3_ with a fixed angle between the axis *M*_1_*M*_2_ and the center axis, let the intersection points between the line segment and the axis be *M*_31_(*x*_31_, *y*_31_, *z*_31_); they should satisfy the following limits:

20 $$ \Big\{{\displaystyle \begin{array}{c}\frac{\overrightarrow{M_{31}{M}_3}\cdot \overrightarrow{M_2{M}_1}}{\mid \overrightarrow{M_{31}{M}_3}\mid \ast \mid \overrightarrow{M_2{M}_1}\mid }=\frac{\sqrt{2}}{2}\\ {}\mid \overrightarrow{M_{31}{M}_3}\mid =\mid \overrightarrow{M_3{M}_{1/2}}\mid \\ {}{x}_{31}={x}_1+\frac{\mid \overrightarrow{M_3{M}_{1/2}}\mid +\mid \overrightarrow{M_1{M}_{1/2}}\mid }{\mid \overrightarrow{M_1{M}_2}\mid}\left({x}_2-{x}_1\right)\\ {}{y}_{31}={y}_1+\frac{\mid \overrightarrow{M_3{M}_{1/2}}\mid +\mid \overrightarrow{M_1{M}_{1/2}}\mid }{\mid \overrightarrow{M_1{M}_2}\mid}\left({y}_2-{y}_1\right)\\ {}{z}_{31}={z}_1+\frac{\mid \overrightarrow{M_3{M}_{1/2}}\mid +\mid \overrightarrow{M_1{M}_{1/2}}\mid }{\mid \overrightarrow{M_1{M}_2}\mid}\left({z}_2-{z}_1\right)\end{array}} $$

We obtain $$ \overrightarrow{M_{31}{M}_3}=\left({x}_3-{x}_{31},{y}_3-{y}_{31},{z}_3-{z}_{31}\right)=\left({m}_1,{n}_1,{k}_1\right) $$_._

Because the angle between two subtubes and the angle between the subtube and the main tube are fixed values, we can calculate the other two direction vectors of the subtubes based on the geometrical relationship.

21 $$ \overrightarrow{B_1^2}=\overrightarrow{M_{31}{M}_{1/2}}+\frac{1}{2}\overrightarrow{M_3{M}_4}+\frac{\sqrt{3}}{2}\left(\overrightarrow{M_3{M}_{31}}\times \overrightarrow{M_{31}{M}_{1/2}}\right)=\left({m}_2,{n}_2,{k}_2\right) $$

22 $$ \overrightarrow{B_1^3}=\overrightarrow{M_{31}{M}_{1/2}}+\frac{1}{2}\overrightarrow{M_3{M}_4}+\frac{\sqrt{3}}{2}\left(\overrightarrow{M_{1/2}{M}_{31}}\times \overrightarrow{M_{31}{M}_3}\right)=\left({m}_3,{n}_3,{k}_3\right) $$

The parametric equation of the three subtubes is as follows.

23 $$ \Big\{{\displaystyle \begin{array}{c}x={x}_{B_i}+{m}_jt\\ {}y={y}_{B_i}+{n}_jt\\ {}z={z}_{B_i}+{k}_jt\end{array}},\mathrm{where}\ i=1,2,3;j=1,2,3 $$

There are three cases for the direction vector of each subtube; let the coordinates of the subtube and the boundary of the hematoma be *B*_11_, *B*_12_, *B*_13_; *B*_21_, *B*_22_, *B*_23_; *B*_31_, *B*_32_, *B*_33._

24 $$ {d}_{B_i}=\max \left(|{B}_1{B}_{1i}|+|{B}_2{B}_{2j}|+|{B}_3{B}_{3k}|\right),\kern0.5em \Big\{{\displaystyle \begin{array}{c}i,j,k=1,2,3\\ {}i\ne j\ne k\end{array}} $$

When we find the maximum value ${d}_{B_i}$, the coordinates of the bifurcation subtube probe in hematoma can be obtained as $\frac{B_1{B}_{1i}}{2}$, $\frac{B_2{B}_{2j}}{2}$, and $\frac{B_3{B}_{3k}}{2}$.

## Results and discussion

**Fig. 5:**
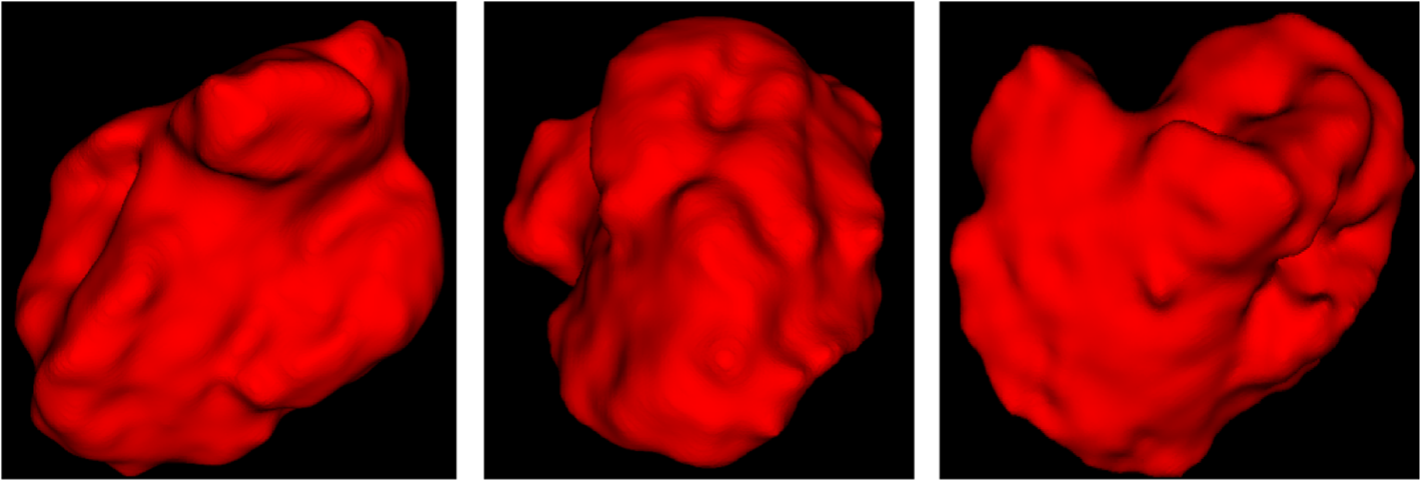
Three-dimensional intracranial hematoma model

According to the CT data, the hematoma area was segmented, and an independent three-dimensional diagram was obtained, as shown in Fig. 5.

**Fig. 6:**
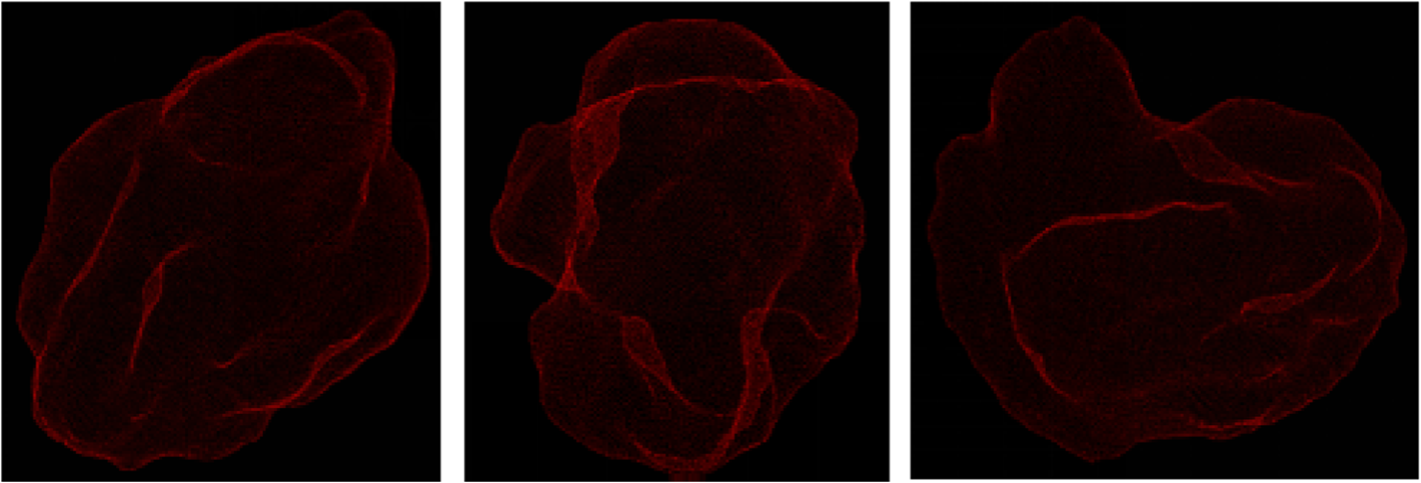
The point cloud model

**Fig. 7:**
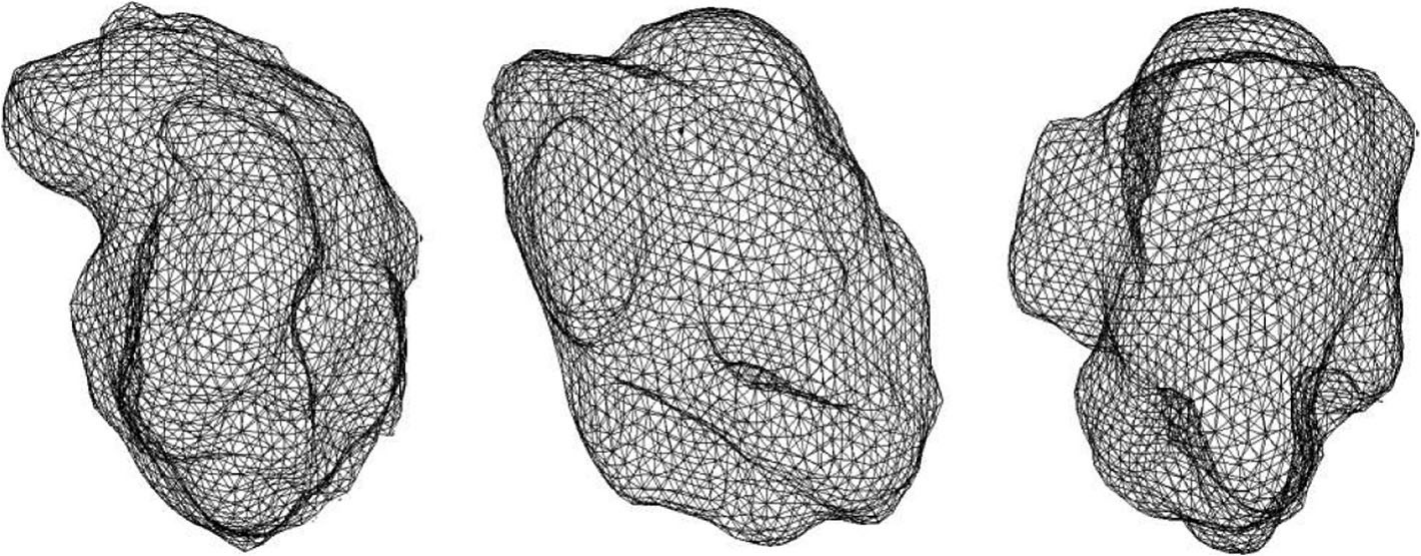
Schematic diagram of triangulation of the hematoma in STL format

The STL format of a three-dimensional point cloud is obtained by hematoma modeling. Figure 6 shows the three-dimensional point cloud diagram in the hematoma area, and Fig. 7 shows the triangulation diagram of this area [24].

**Fig. 8:**
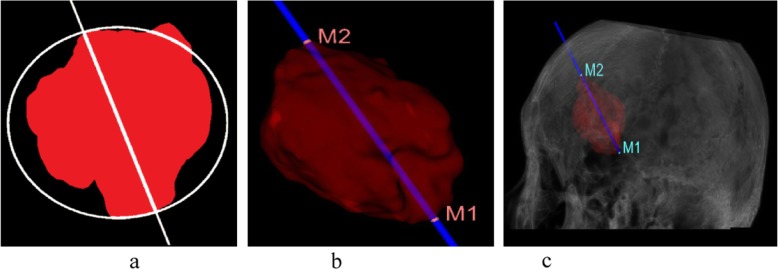
Optimal external central axis of the hematoma. **a** Two-dimensional projection; **b** Three-dimensional projection; and **c** The exact position in skull

In this case, the point cloud search arithmetic method for modified adaptive weighted particle swarm optimization to search the hematoma region uses 81,226 point-chains. The coordinates of the intersection between the central axis and the boundary of the hematoma are *M*_1_(*x*_1_, *y*_1_, *z*_1_) and *M*_2_(*x*_2_, *y*_2_, *z*_2_). The optimal external central axis of the hematoma is shown in Fig. 8. The intersection points of the central axis and the boundary of the hematoma are *M*_1_(28.555, 9.623, 7.123) and *M*_2_(43.384, 21.295, 48.887)_._

Therefore, the midpoint coordinates of the center axis *M*_1_*M*_2_ are *M*_1/2_(35.970, 5.836, 28.010), and the distance from *M*_1_ to *M*_2_ is *d*_12_ =  ∣ *M*_1_*M*_2_ ∣  ≈ 54.0377. Figure 8 is optimal external central axis of the hematoma.

The route planning for the puncture operation is as follows:

Because $\frac{d_{B_3}-{d}_{B_2}}{2}+{d}_{B_2}=35<{d}_{12}\approx 54.0377$, it is suggested that doctors choose three subtube drainage tubes when performing puncture operations. From the coordinate of the center axis, the plane equation through *M*_3_*M*_4_ can be obtained as

$$ 14.82919\left(x-35.969525\right)+30.91862\left(y-5.83595\right)+71.763726\left(z-28.009754\right)=0. $$

According to the point cloud search arithmetic method for modified adaptive weighted particle swarm optimization, we obtain the coordinates as *M*_3_(26.201.451, 18.457) and *M*_4_(36.353, −7.797, 37.816), and the distance from *M*_3_ to *M*_4_ is *d*_34_ = 38.134751.

Through the above algorithm process, the coordinates of the three bifurcation points can be calculated

$$ {B}_1\left(35.18971256,4.219077228,25.81685749\right), $$

$$ {B}_2\left(37.93409287,9.939157835,33.54479557\right), $$

$$ {B}_3\left(40.67847319,15.65923845,41.27273366\right). $$

The three direction vectors of the subtube are as follows:

$$ \overrightarrow{M_{31}{M}_3}=\left(-0.296512566,-24.3104924,-4.838389244\right), $$

$$ \overrightarrow{B_1^2}=\left(3.125640267,-0.881458887,-10.79916805\right), $$

$$ \overrightarrow{B_1^3}=\left(-6.200200368,\kern0.5em -1.216682803,-3.497583415\right). $$

There are three cases for the direction vector of each subtube; let the coordinates of the subtube and the boundary of the hematoma be *B*_1, 1_, *B*_1, 2_, *B*_1, 3_; *B*_2, 1_, *B*_2, 2_, *B*_2, 3_; *B*_3, 1_, *B*_3, 2_, *B*_3, 3_. The maximum value from the bifurcation point to the boundary of the hematoma is

$$ {d}_{B_{ii}}=\max \left(|{B}_1{B}_{1,i}|+|{B}_2{B}_{2,j}|+|{B}_3{B}_{3,k}|\right),\Big\{{\displaystyle \begin{array}{c}i,j,k=1,2,3\\ {}i\ne j\ne k\end{array}}. $$

The coordinates of the bifurcation probe to reach the hematoma area are

$$ {B}_{11}=\left(35.11854955,-1.615440948,24.6556440\right), $$

$$ {B}_{22}=\left(39.80947703,9.410282503,27.0652947\right), $$

$$ {B}_{33}=\left(38.19839304,15.17256533,39.8737003\right). $$

**Fig. 9:**
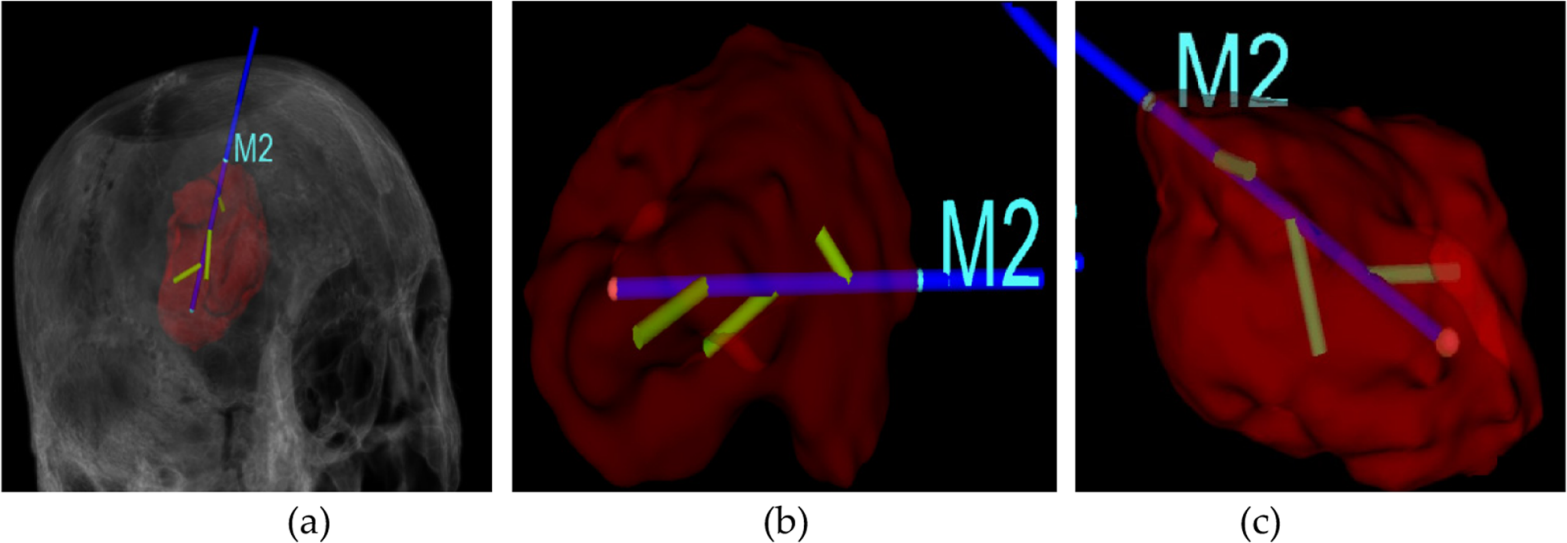
Route planning. *M*_2_ denotes the accurate position of the puncture point

Figure 9 is a schematic diagram of the path planning process.

In this study, 3D Slicer software was used to reconstruct a three-dimensional model of an intracranial hematoma, and an improved algorithm based on clustering and adaptive particle swarm optimization to extract the optimal external central axis of the hematoma was proposed. According to the characteristics of the drainage tube and the specific morphology of the intracranial hematoma, optimal path planning under ideal conditions was performed, and the preoperative simulation was provided, which will be useful in clinical medicine.

The bifurcation localization algorithm of the drainage tube proposed in this study assumes an ideal state. It is necessary to consider the distribution of peripheral nerves and blood vessels and the specific location of the hematoma in the brain to apply this method to clinical medicine. The focus of subsequent research work will be to optimize the algorithm by considering the above factors, to implement the diffusion model and simulation of the drug injection, to plan the optimal surgical project, and to provide a dynamic simulation before operation.

## Data Availability

The datasets used and/or analysed during the current study are not publicly available due to personal privacy but are available from the corresponding author on reasonable request. Supplementary Materials: The following are available online at www.mdpi.com/xxx/s1, Figures, Tables .

## Abbreviations

CT: Computerized tomography
ROI: Region of interest
